# Saharan dust and giant quartz particle transport towards Iceland

**DOI:** 10.1038/s41598-021-91481-z

**Published:** 2021-06-04

**Authors:** György Varga, Pavla Dagsson-Waldhauserová, Fruzsina Gresina, Agusta Helgadottir

**Affiliations:** 1 Geographical Institute, Research Centre for Astronomy and Earth Sciences, Budapest, Hungary; 2 Faculty of Environmental and Forest Sciences, Agricultural University of Iceland, Reykjavik, Iceland; 3 Faculty of Environmental Sciences, Department of Ecology, Czech University of Life Sciences, Prague, Czech Republic; 4 Institute of Geography and Earth Sciences, Faculty of Science, Budapest, Hungary; 5 Soil Conservation Service of Iceland, Hella, Iceland

**Keywords:** Atmospheric dynamics, Climate-change impacts

## Abstract

Mineral dust emissions from Saharan sources have an impact on the atmospheric environment and sedimentary units in distant regions. Here, we present the first systematic observations of long-range Saharan dust transport towards Iceland. Fifteen Saharan dust episodes were identified to have occurred between 2008 and 2020 based on aerosol optical depth data, backward trajectories and numerical models. Icelandic samples from the local dust sources were compared with deposited dust from two severe Saharan dust events in terms of their granulometric and mineralogical characteristics. The episodes were associated with enhanced meridional atmospheric flow patterns driven by unusual meandering jets. Strong winds were able to carry large Saharan quartz particles (> 100 µm) towards Iceland. Our results confirm the atmospheric pathways of Saharan dust towards the Arctic, and identify new northward meridional long-ranged transport of giant dust particles from the Sahara, including the first evidence of their deposition in Iceland as previously predicted by models.

## Introduction

Mineral dust particles emitted from arid–semiarid areas are vital active contributors to several meteorological and climatic processes as well as other environmental and health processes^1–10^. Billions of tons of mineral dust particles are lifted and transported from the source areas of North Africa, the Middle East, Middle and Eastern Asian deserts, Australia, and arid regions of North and South America every year^7,11–13^. In particular, Saharan dust source areas are responsible for an estimated 50–70% of global windblown mineral dust emissions^14,15^. The mass of fine-grained mineral particles emitted annually from the African continent can reach 1–2 × 10^9^ t.

Active dust sources are also located farther away from the typical low-latitude source areas. High-latitude dust (HLD) sources that produce at least 10^8^ t of fine dust are situated in Iceland, Greenland, Alaska, Canada, Patagonia, Antarctica, and New Zealand among other areas^16^. Iceland is one of the most important HLD source regions and is the largest desert area of Europe, with approximately 44,000 km^2^ of desert areas^17^. As the largest European and Arctic desert, Iceland is an active high-latitude dust source^16,17^. The arid, largely barren volcanic island is predominantly covered by volcanic, volcanoclastic, and aeolian deposits (sand sheets, sand dunes, and loess deposits). Volcanoclastic sandy deserts, glacio-fluvial lowland and highland plains, and beach areas in Iceland are primary sources of fine-grained, unconsolidated sediments. Approximately 135 dust days are observed annually, placing Iceland between the dustiest places on Earth^18–20^. Approximately 30–40 × 10^6^ t of mineral dust is emitted every year from Icelandic source areas into the atmosphere^16,21^.

Both local dust and dust transported from distant areas have major impacts on climatic and environmental processes in fragile high-latitude regions^17,22–24^. In addition to the direct effect on incoming shortwave and outgoing longwave radiation via absorption, scattering, and diffraction, atmospheric dust plays a notable role in cloud condensation and ice nuclei formation^23,25–29^. Furthermore, a high amount of bioavailable iron in suspended dust can have a major effect on high-latitude oceanic biogeochemical cycles and productivity^21,30–34^, and hence on the carbon cycle. Dark, basaltic dust particles from Iceland significantly decrease the albedo of snow- and ice-covered regions^24,35–38^. As a fundamental nutrient source for algae in ice, dust can enhance ice melting^39–41^. To better understand the high-latitude dust cycle, information on the detailed granulometric and mineral properties of dust material is needed. Dust particles are transported long distances from the island, and have been identified in Serbia, Svalbard, Central Greenland, and the British Isles^42–47^.

Similarly, as HLD can be transported and deposited thousands of kilometres from high-latitude regions^46^, low-latitude dust should be taken into account at high-latitudes. Dust from North African sources can reach distant regions located tens of thousands of kilometres from the sources, such as the Americas, the Caribbean, Northern Europe, and Greenland^47–58^. Saharan dust sources are known to produce material contributing to fine-grained dust deposition in the Arctic region^59^ [e.g. Greenland^50,60^, Scandinavia^43,61^, and Iceland (this study)]. No known study has reported the presence of Saharan dust in Iceland, although satellite images, local visual observations, and dust models have indicated that such events do occur^1^. Enhanced meridional atmospheric flow patterns provide suitable conditions for the long-range, poleward transport of Saharan dust^50,60,62^.

Pleistocene glacial dust deposition in the Arctic has been extensively studied in ice cores from Greenland, and potential dust sources have been reported for Asia, North America, and the Sahara^63–67^. Several studies have eliminated North Africa as a probable dust source region based on clay mineralogical and Sr/Nd isotopic considerations^63,64^. Svensson et al.^64^ emphasised that dust found in an ice core from Greenland could have originated from Saharan source areas that have not yet been sampled. Újvári et al.^66^ discussed the inability of the widely applied methods to discriminate among different potential sources.

The objectives of this study are to (1) provide evidence of the long-range transport of Saharan dust to Iceland and estimate the frequency of such events; (2) provide meteorological and sedimentary information on Saharan dust transport towards higher latitudes; (3) discuss the possibility of coarse mineral dust transport from North Africa to Iceland; (4) provide mineralogical information on local Icelandic dust to be distinguished from Saharan dust (and to exclude to potential local contamination of the deposited dust material); and (5) discuss for and against arguments regarding giant Saharan quartz particles in Iceland.

## Results

**Figure 1:**
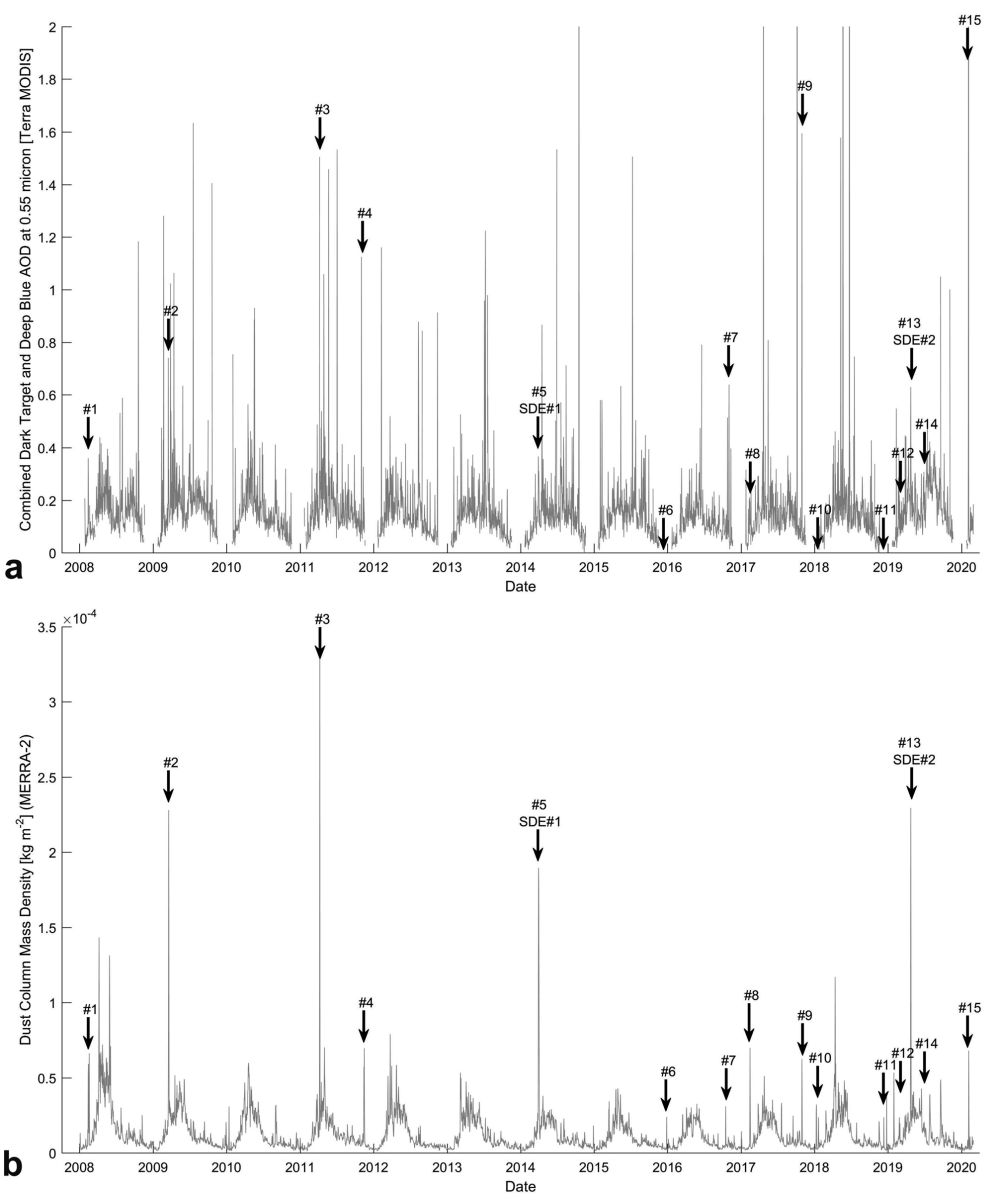
Aerosol optical depth data (**a**) and MERRA-2 dust column mass density data (**b**) of the study area and the identified Saharan dust events (#1–#15).

Fifteen (#1–15) Saharan dust events were identified in Iceland between 1 January 2008 and 29 February 2020. A systematic combined analysis of Moderate Resolution Imaging Spectroradiometer (MODIS) aerosol optical depth (AOD) data and Hybrid Single-Particle Lagrangian Integrated Trajectory (HYSPLIT) data resulted in the identification of nine of these fifteen dust events. These nine episodes matched distinct AOD peaks (Fig. 1). The remaining six episodes were determined based on the numerical simulations of the NASA’s Modern-Era Retrospective analysis for Research and Applications, Version 2 (MERRA-2) and Barcelona Supercomputing Center (BSC), and verified using satellite images and backward trajectories of the HYSPLIT model.

All 15 dust episodes were independently examined by analyses of Cloud-Aerosol Lidar and Infrared Pathfinder Satellite Observation (CALIPSO) vertical aerosol subtype profiles. The presence of dust over Iceland was confirmed by the lidar data for all 15 episodes. Among the events, two rare wet depositional episodes were detected; in these cases, the sampled Saharan dust materials were analysed using automated static image analysis.

### Meteorology of the Saharan dust events #1–15

**Figure 2:**
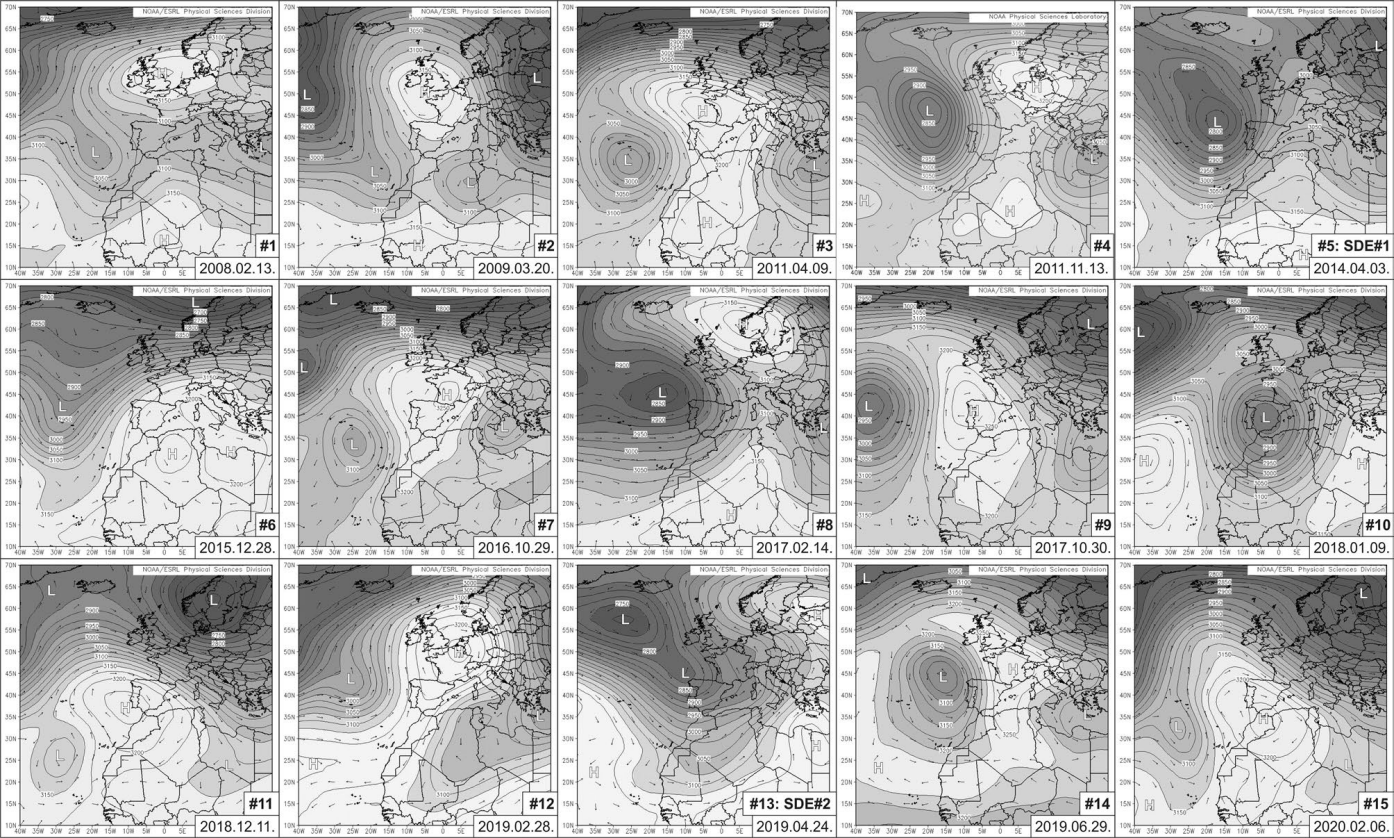
Synoptic meteorological situation of the identified Saharan dust events towards Iceland (geopotential height and wind vectors at 700 hPa).

Based on geopotential height maps and wind vectors at 700 hPa, two different synoptic meteorological situations were distinguished (Fig. 2). The Omega block-like pattern determined the Saharan dust transport of nine episodes. The high-pressure centre of the blocking system was located over Western–South-western Europe. The steepest pressure gradients and connected strongest flows were situated at the southwest side of the anticyclonic system.

Synoptic patterns of the remaining six events were associated with a low-pressure system situated over (or close) to the Iberian Peninsula. The highest wind speeds were observed at the foreside of the stationary cyclone. Central and Eastern European blocking systems were also responsible for the development of the extended meridional atmospheric ‘conveyor belt’.

**Figure 3:**
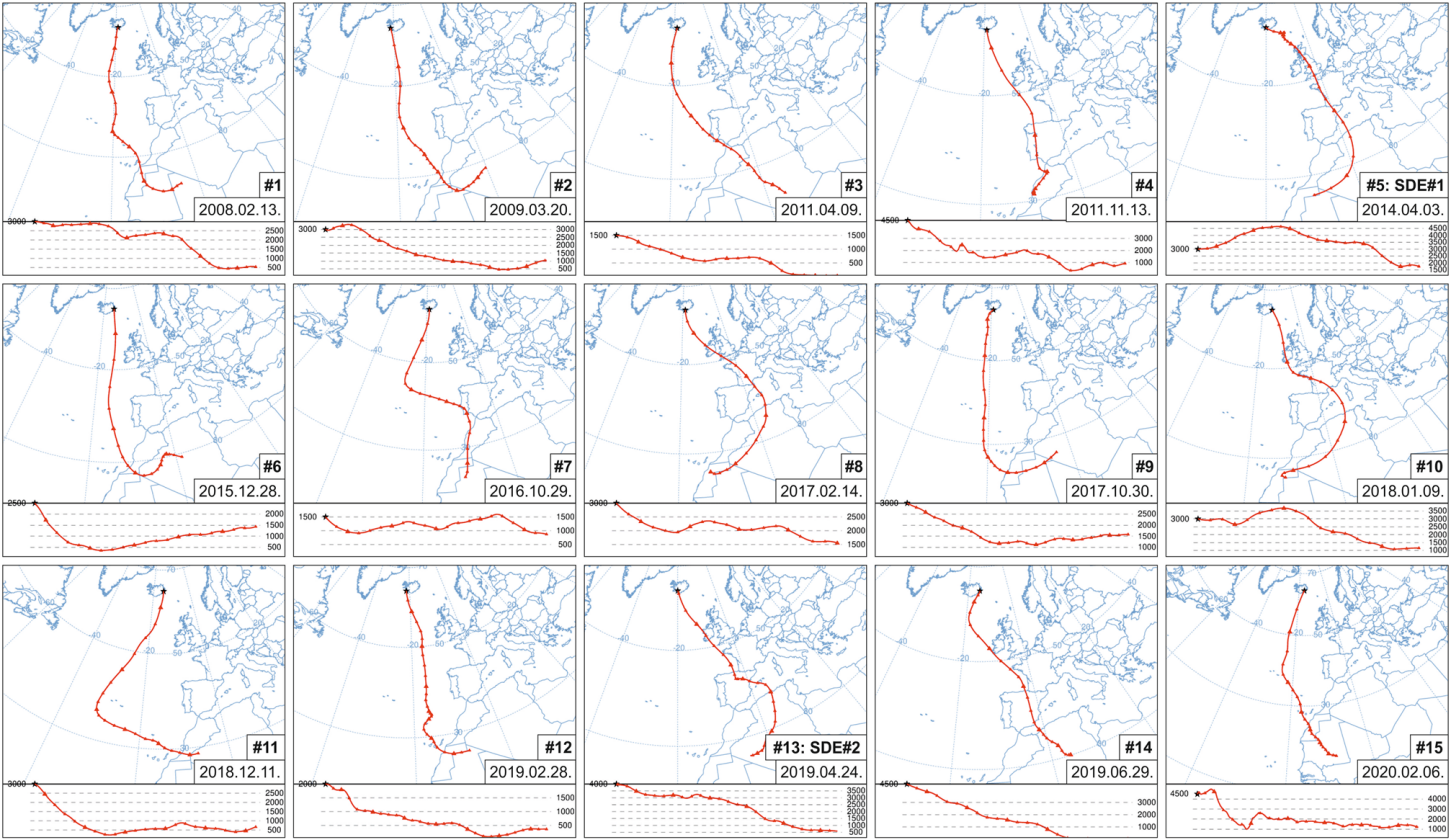
Backward trajectories (HYSPLIT model) of the identified Saharan dust events in Iceland.

Dust transport pathways were mapped using back trajectories calculated at different heights from Iceland (Fig. 3). The two main types of synoptic patterns defined two specific transport routes: one directly over the Atlantic Ocean and another over Europe (Spain, France, and the British Isles). Typical trajectory lengths were ~ 4500 to 5000 km, with possible source areas of the dust material located in Northwest Africa. Daily surface observations of the United States Naval Research Laboratory (Monterey aerosol page: https://www.nrlmry.navy.mil/aerosol/#aerosolobservations) allowed a more detailed source appointment. The most important dust sources can be regarded as NW African dust hot spots^68^, like (1) the ancient lakebed deposits of the Taoudenni Basin, (2) large alluvial fans and extensive wadi-system on the western and north-western slopes of the Hoggar Mountains (Ahaggar Plateau and foothills), (3) intramountain sediments of the Atlas Mountains, and (4) coastal sebkhas of Western Africa.

### Saharan dust deposition in Iceland (events #5 and #13 marked as SDE #1 and SDE #2 in Figs. 1, 2, 3)

**Figure 4:**
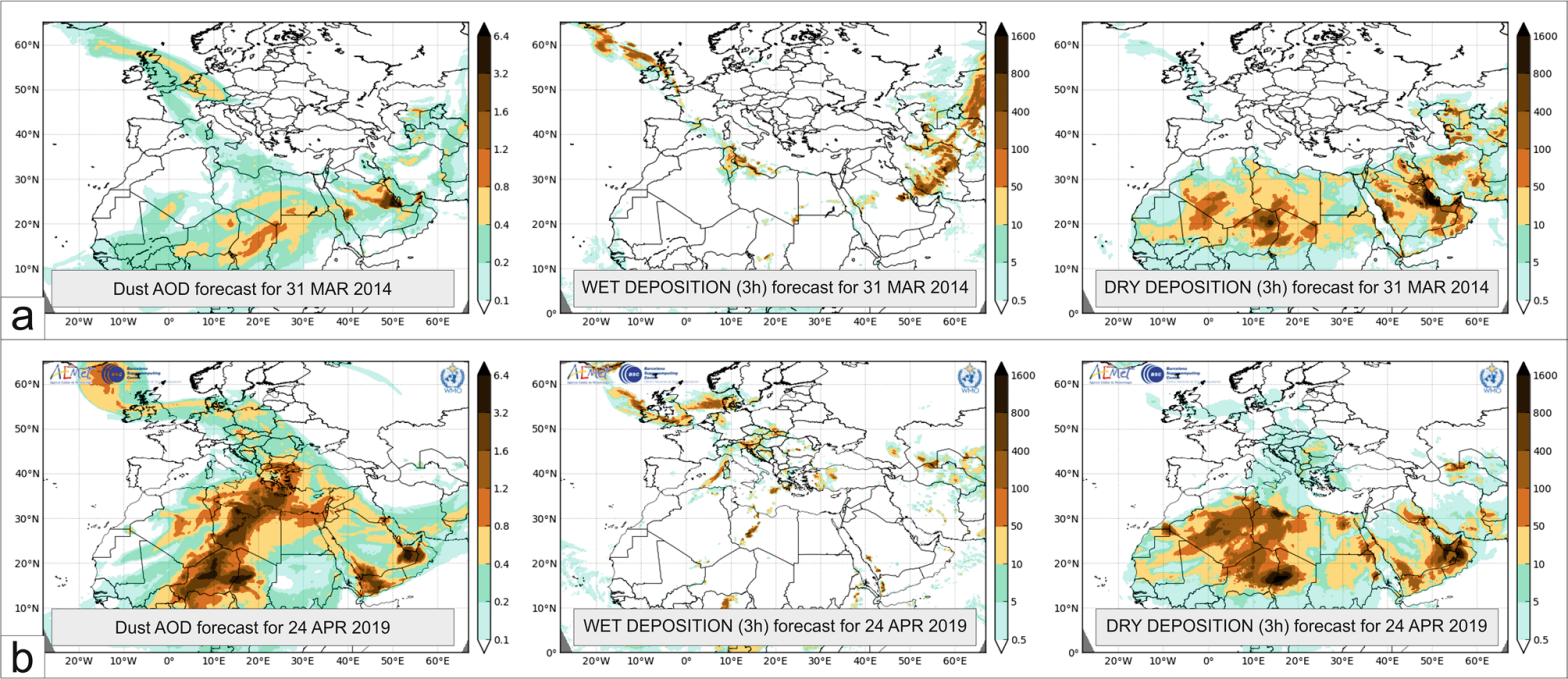
Numerical dust forecast maps (aerosol optical depth (AOD]; 3 h wet and dry deposition) of two dust depositional episodes: SDE #1 and SDE #2.

The general meteorological background and related transport routes, as well as the possible source areas of the two depositional events, in Iceland were very similar. The first deposition event (SDE #1) occurred during the last days of March 2014, when a low-pressure system governed the atmospheric patterns of the eastern basin of the North Atlantic. The cyclonal flow of the system reached Northwest Africa, where the steep pressure gradient between the mid-latitude low and Saharan highs triggered sand and dust storms. A high amount of lofted mineral dust was transported northwards along the eastern flank of the stationary atmospheric trough system blocked by the Central European ridge. Dust material reached the atmosphere of France on 28 March 2014 and consequently Great Britain and Ireland on 29 March 2014. Intense wet deposition of dusty material and ‘blood rain’ events were reported for the affected areas^58,69^. Atmospheric dust was deposited in Iceland on 3 April 2014, as shown by wet deposition in the Icelandic domain in the dust numerical simulations of the NMMB/BSC (Non-hydrostatic Multiscale Model/Barcelona Supercomputing Center) (Fig. 4a). Precipitation did not affect all parts of Iceland, and samples collected in Keldnaholt were dry. Particulate matter (PM_10_) concentrations from available stations in Reykjavik were twice as high as the annual mean, with hourly concentrations of > 50 µg m^−3^ at the background station (2014 annual mean: 8 µg m^−3^; daily mean: 18 µg m^−3^) and > 180 µg m^−3^ at an urban station (2014 annual mean: 21 µg m^−3^; daily mean: 51 µg m^−3^).

The second deposition event (SDE #2) occurred on 25 April 2019. The deep cut-off low on 20 April 2019 (closed circulation system from an upper-level trough in the antecedent days) determined the synoptic situation of North Africa, and the cyclonal flow lofted enormous mass of desert dust into the atmosphere. In the following few days, the atmosphere from Africa through Crete over Europe to Iceland was loaded with an enormous amount of Saharan dust, reaching a PM_10_ concentration of > 150 µg m^−3^^70^. The blocking mechanism of an Eastern European anticyclone was responsible for the stationary behaviour of the atmospheric system. Saharan dust arrived over Iceland on 24–25 April via a transport route (from the southern forelands of the Atlas Mountains through the Iberian Peninsula and across the British Isles towards Iceland). The pathway was very similar to that of SDE #1.

The forecasts of the BSC for dust load and wet deposition clearly showed the north(west)ward Saharan dust transport governed by the previously presented meteorological flow patterns (Fig. 4b). Mineral dust material from the foreside of the Atlas Mountains reached high latitudes across Western and North-western Europe over a 4-day period (the exact amount of deposited dust can only be roughly estimated from the models). Particulate matter (PM_10_) concentrations from available stations in Reykjavik were 4-times higher than the annual mean, with hourly concentrations of > 50 µg m^−3^ at the background station (2019 annual mean: 8 µg m^−3^; daily mean: 33 µg m^−3^) and > 270 µg m^−3^ at the urban station (2019 annual mean: 15 µg m^−3^; daily mean: 73 µg m^−3^). Additionally, dust measurements from Akureyri in North Iceland are available when the PM_10_ concentration increased to at least 3-times the 2019 annual mean of 16 µg m^−3^. Dust deposition started in Akureyri on 24 April and resulted in a mean concentration of 62 µg m^−3^ with a maximum hourly concentration of > 220 µg m^−3^.

### Granulometry of SDE samples compared to local Icelandic dust source samples

**Figure 5:**
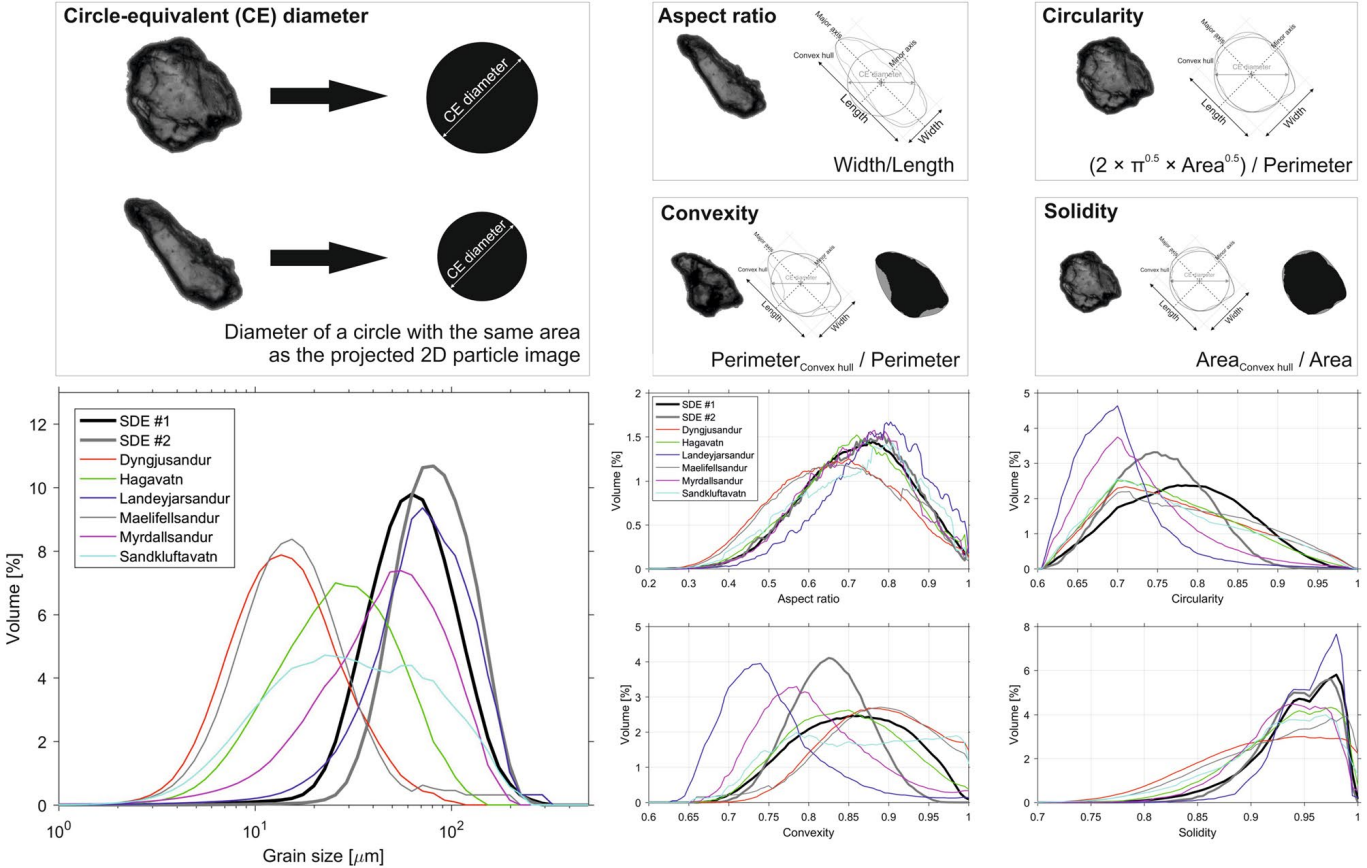
Distribution curves of the grain size and shape of bulk samples from SDEs and Icelandic source areas.

A detailed laboratory analysis of the samples was necessary to distinguish between local and remotely transported particulate matter and to exclude possible local dust addition and contamination of the deposited dust material. An automated static optical image analysis of several thousand individual particles was performed to obtain a robust granulometric characterisation based on the distribution curves of grain size and shape (Fig. 5). The grain size distribution curves of deposited material during the SDEs in Iceland showed clear unimodal distributions, which fell predominantly into the coarse silt (20–62.5 µm) and (very) fine sand (62.5–(125)–250 µm) particle size fractions. The modal grain sizes, median diameters, and mean diameters D[3,4] were 62.9 µm and 81.2 µm, 65.7 µm and 87.0 µm, and 70.6 µm and 89.3 µm for SDE #1 and SDE #2, respectively.

The circle-equivalent (CE) diameters of bulk samples from Icelandic sources were diverse. The grain sizes of samples from Landeyjarsandur and Mýrdalssandur fell into the coarse silt/fine sand fractions, whereas the particles of other samples were much smaller, especially the samples of very fine-grained deposits from Dyngjusandur and Mælifellssandur.

According to previous studies (e.g. Varga et al.^71^), the grain shape parameters of automated static optical image analysis are size-dependent variables (direct comparison of shape parameters of particles with significantly different sizes do not provide realistic results). Thus, particles in the size ranges of the SDE samples (i.e. 20–250 µm) were selected to create adequate conditions for a representative particle shape comparison. The SDE samples exhibited a more convex and circular particle shape compared to the local Icelandic samples. The general patterns of the aspect ratio distribution curves were similar, but the solidity curves clearly indicated major differences among the different sample groups. The SDE samples and Landeyjarsandur (a sandy beach that is not directly affected by glacial processes) sample presented similar curves.

**Figure 6:**
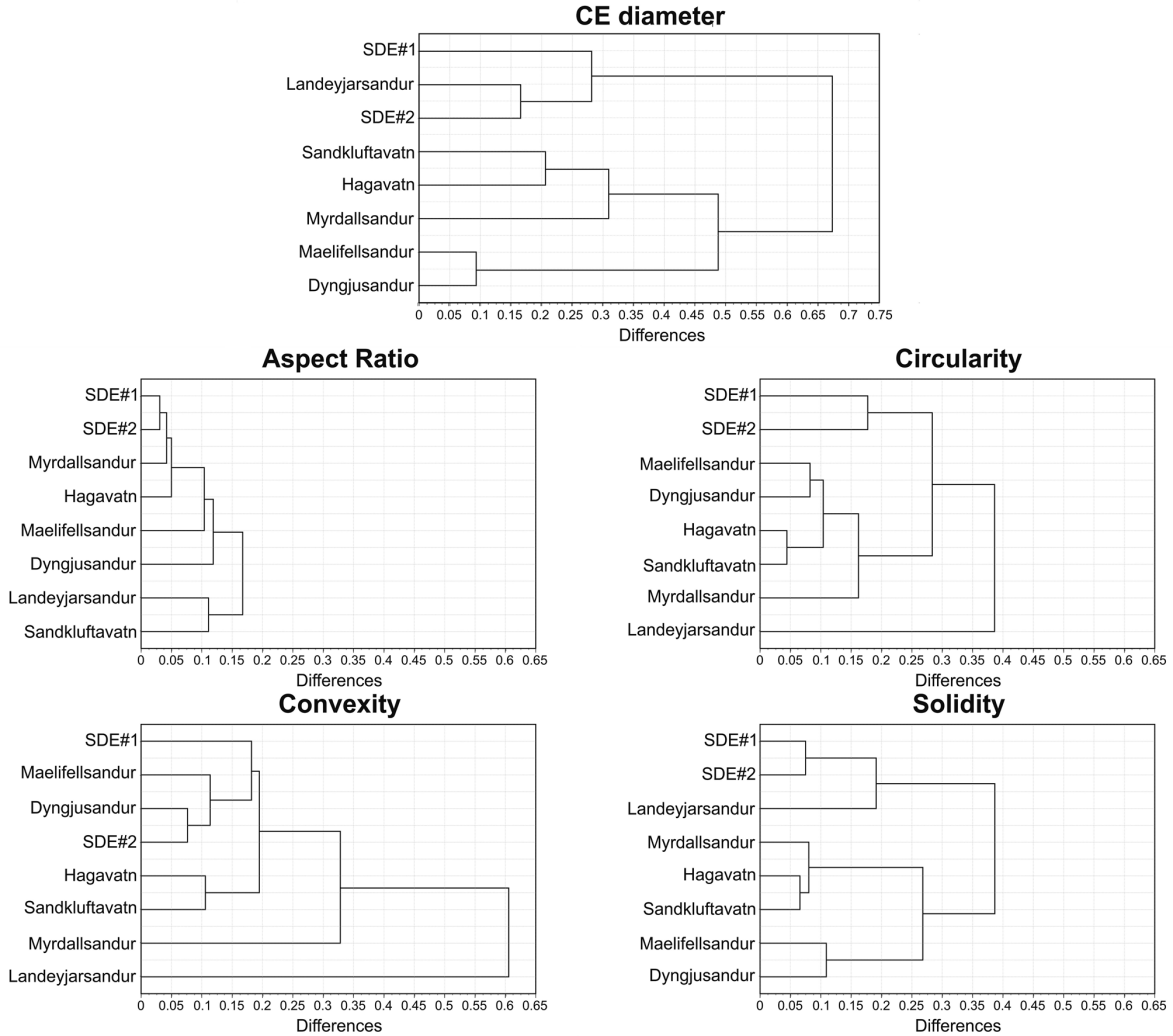
Hierarchical cluster trees of size and shape parameters of SDE samples and local surface samples.

Cluster analyses of different size and shape parameters were also performed (Fig. 6). Based on the grain size distribution and solidity shape parameter, the SDE samples were clustered close to Landeyjarsandur, whereas other Icelandic dust hotspots consisted of significantly finer grains. The analyses of other major shape parameters (aspect ratio, convexity, and circularity) resulted in various trends for different materials, with the solidity parameters showing a similar clustering for the same particle size-based groups of material. In general, the SDE samples were closely clustered, whereas the Icelandic samples were not; however, there were similarities between the samples from Dyngjusandur and Mælifellssandur. Irregular circularity and convexity of particles were typical characteristics of material from Landeyjarsandur compared to other Icelandic hotspots.

Raman spectroscopy also confirmed the differences between the materials from SDEs and local sources. Due to the high percentage of particles with nonapplicable Raman signals, robust quantitative analyses could not be made. Our general findings, however, clearly showed that Icelandic surface samples obviously reflected the local basaltic environment, and that no quartz particles were found in these samples. Quartz particles, including particles larger than 100 µm, were identified in large numbers in both SDE samples.

## Discussion

### Enhanced atmospheric meridionality and climate change

The surface temperature anomaly during the two SDEs compared to the climatological mean for the period from 1981 to 2010 (by applying the reanalysis data of the National Center for Environmental Protection (NCEP) and National Center for Atmospheric Research (NCAR)^72^) was 3.1 °C. Enhanced meridionality of the upper-level atmospheric flow was the main synoptic driver of the warm incursions and the identified Saharan dust events. The importance of a more meandering polar jet and associated meridional flow patterns during the formation of the North African cyclone and poleward dust transport was discussed by Francis et al. (2018). According to the authors’ findings, the intense warming of Arctic regions reduces the temperature difference between the high- and mid-/low-latitude areas, thus leading to increasing planetary wave amplitudes and more meridional flow patterns at high altitudes^73^.

The southward propagation of the upper-level atmospheric trough and the orographic blocking of the Atlas Mountains play vital roles in the formation of severe surface wind storms and dust entrainment in the northwest regions of the Sahara^74^. The area of these Northwest African dust hotspots was identified as the main source of the discussed Icelandic dust events; hence, the Icelandic samples were collected approximately 4000–4500 km from their potential Saharan sources.

### Long-range meridional transport of giant Saharan dust

In the recent years several studies were published on long-ranged giant dust transport. It has been shown that coarse particles with a diameter of > 30 µm and giant particles with a diameter of > 100 µm are capable of long-range transport within the Saharan air layer^75–79^. Previous studies focused on the zonal (east-to-west) transport of giant Saharan dust, whereas meridional (south-to-north) transport towards higher latitudes has only been mentioned in some recent papers^62,80^. In particular, aerosol studies on the long-range transport of coarse and giant particles to the Arctic are generally lacking^81^. Here, we provide the first evidence of giant Saharan particles deposited in Iceland. Our results confirm the existence of established atmospheric pathways of Saharan dust towards the Arctic, with Saharan dust contributing ~ 32% of the total atmospheric dust load in the Arctic^59^.

The long-range transport of sand-sized and/or giant dust particles, however, is still an open question and little is known about the key drivers of transport mechanisms. Several possible transport mechanisms have been suggested, including strong winds, enhanced atmospheric vertical mixing due to strong turbulence via vertical up-flow of thunderstorms and tropical cyclones, and triboelectrification of dust particles.

Several studies have reported that dust deposition was poorly simulated by climate models and other numerical simulations^82,83^, primarily due to the false parametrization of the grain size of dust particles. There is a general bias in the size distribution of atmospheric dust, which is skewed towards finer size fractions^79,82,84,85^. Underestimation of the transport and deposition of these coarse mineral particles leads to further biases in the derived climatic effects (and other environmental effects of) aeolian dust (e.g. radiative forcing, which possibly causes net atmospheric warming, and a role in biogeochemical cycles and soil formation). So, the presented size data also has a climatic relevance as the interpretation questions of coarse-sized windblown dust are standing in the focal point of recent studies. Climatic impacts of mineral dust are also sensitive to particle size and shape. Radiative effects of coarse mineral grains in the shortwave spectrum is determined by the lower single scattering albedo compared to finer particles, causing more absorption of solar radiation and so, heating of the atmosphere^86,87^. Ryder et al.^87^ reported an increased atmospheric heating by up to a factor of three when as a result of calculated single scattering albedo dropping from 0.92 to 0.8 when larger particles were taken into account. Kalashnikova and Sokolik^88^ showed that irregular shape character of windblown mineral particles also have an effect on radiative properties via larger surface-to-volume ratio relative to that of an equal volume sphere. According to the calculations of Kok et al.^86^, average global dust extinction efficiency results are underestimated by 20–60% for larger than 1 micron particles.

### Differences of granulometric properties of Icelandic and SDE samples

Various size and shape parameters of the (1) local Icelandic samples, and (2) collected mineral material of the SDEs showed different characteristics. These differences in the two sediment groups were also confirmed by cluster analysis. Clustering of the shape parameters of the SDE and local surface samples showed an outlying nature of the particles from the washout samples. The washout samples mainly contained coarse silt and fine sand-sized mineral dust material, which could be clearly distinguished by their different size and shape parameters in comparison to the samples from local source areas^89–91^. The more circular and convex shape along with higher solidity values indicated a more mature nature of the SDE particles, which was significantly different to the nature of the fresh volcanoclastic deposits of Iceland. We infer that these granulometric differences could relate to (1) the relatively young geological evolution history of local samples, and (2) the initial shape properties and long-range transport processes of Saharan mineral dust particles.

Similar shape features of far-travelled Saharan dust particles have been reported in other studies^62,80,92^ that applied the same approach and used the same Malvern Morphologi G3-ID device as used in the present study. We are not aware of any other automated static image analysis data of windblown North African mineral dust material (shape data made by other methodological approaches cannot be quantitatively compared).

The well-rounded (but not spherical) shape of coarse silt and fine sand particles (caused by rapid abrasion and edge-rounding) is a known property of windblown deposits; however, sharp edges of finer silt-sized fractions (typical for fine-grained aeolian silts) were also visible on the captured images of individual particles of the SDE samples. Contrary to these properties, the Icelandic samples were much more irregular in shape and dominated by sharp features, even in the case of larger particles^89–91^.

Mineralogical comparisons of Icelandic and Saharan dust have shown that the latter is mainly comprised of quartz and feldspar, while the former primarily consists of volcanic glass^34^. Mineralogical fractionation is not observed in Icelandic dust due to the lack of larger mineral grains such as quartz. It is clear from the detailed laboratory analyses that the local addition of coarse particulate matter to the sampled deposited particles can be excluded.

## Conclusion

In this paper, we presented the first systematic observation, synoptic background, and long-range transport routes of Saharan dust storms towards Iceland. Fifteen Saharan dust events were identified during a 12 year period (1.25 events annually) by applying MODIS satellite products of the NASA Terra Satellite, backward trajectories of the HYSPLIT model, and the numerical model forecasts of NASA’s MERRA-2 and Barcelona Supercomputing Center. Samples of Saharan mineral dust that was deposited in Iceland during two outstanding wet deposition episodes were analysed in detail by using automated static image analysis of several tens of thousands of individual particles. There were clear granulometric differences between the SDE samples and the Icelandic dust source samples, indicating the external nature of the sampled grains and we were able to exclude possible contamination of local dust material. In addition, giant quartz particles (> 100 µm) were found among the deposited material, which confirmed the possible long-range meridional transport of these exceptionally large particles towards the Arctic region. Previous studies on giant particle transport from North Africa were focused on zonal (east to west) dust transport routes. Saharan dust episodes were associated with the enhanced meridionality of high-altitude winds. The meandering flow patterns of jets could be closely related to the increased warming of the Arctic and decreasing temperature difference between high- and low-latitude regions.

## Methods

### Study site for local samples and transported Saharan dust samples

**Figure 7:**
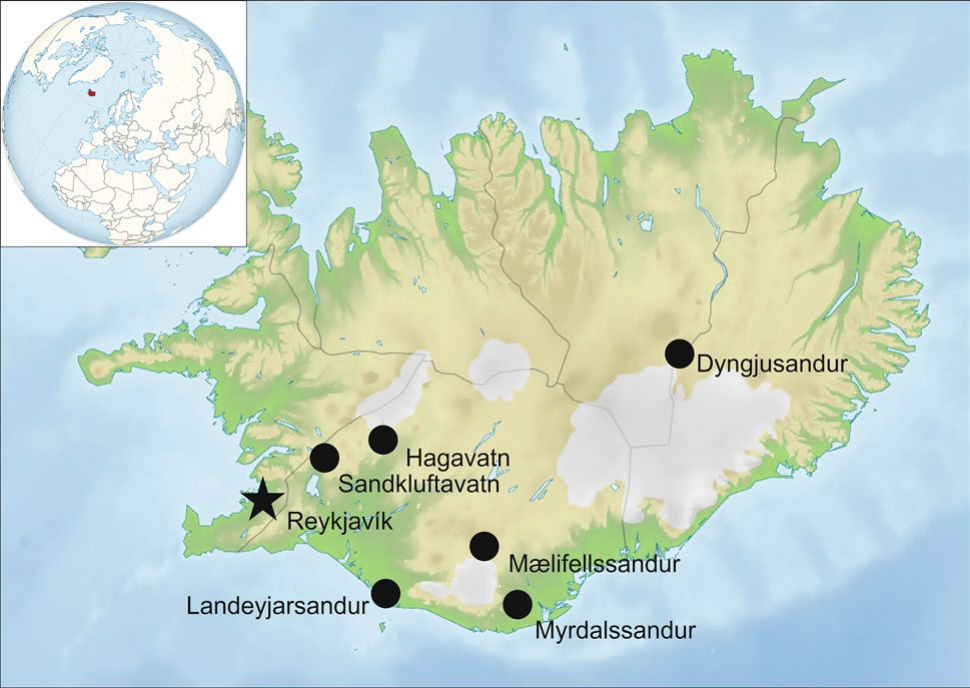
Map of the study area, showing the location of dust hotspots (dots) and the Saharan dust sampling site (star) ( Source of the base maps: https://en.wikipedia.org/wiki/File:Iceland_relief_map.jpg; ).

The surface sedimentary units of six major Icelandic dust hotspots were sampled to obtain the granulometric (grain size and shape) properties of local source materials (Fig. 7).

The Hagavatn dust hotspot is located in the north-eastern part of the Lambahraun plain, and is bordered by moraines. Glacial surges and meltwater release in the outwash plain are responsible for the accumulation of fine-grained deposits in the lake, where the fluctuating water level often leaves loose unconsolidated sediments on the surface. Landeyjarsandur is a floodplain area of the Markarfljót glacial river and sandy beach area. The glacio-fluvial plain of Mælifellssandur is situated to the north of the Mýrdalsjökull glacier, and receives a huge amount of unconsolidated silty material every summer during melting episodes of nearby glacial areas. Mýrdalssandur is located to the southeast of the Mýrdalsjökull glacier, and is also a glacio-fluvial lowland plain under the effect of fluctuating glacial waters and jökulhlaups. Dust is generally transported towards the ocean from this source area. Dyngjusandur is situated in the rain shadow area of the Vatnajökull glacier, and is bordered by Askja (an active volcano) to the north, by the major glacial drainage system of Jökulsá á Fjöllum to the east, and by Vaðalda (a shield volcano). A significant part of the region is covered by an active and continuous sand sheet covering an area of approximately 170 km^2^ (large parts were covered and blocked by Holuhraun lava during 2014–2015) with a thickness of up to 10 m. Fine-grained material originates from the summer floods associated with meltwater from the Vatnajökull glacier, although autumn dust events also occur in some years. Sandkluftavatn is a small lake bed approximately 50 km northeast of Reykjavik.

Samples of Saharan mineral dust that was deposited during two identified SDEs in Iceland were collected at the Agricultural University of Iceland in Keldnaholt, which is in the vicinity of Reykjavik, on 3 April 2014 and 25 April 2019 (a detailed description of each episode is provided in the Results).

### Identification of Saharan dust in Iceland

Persistent cyclonic activity causes frequent cloud obstruction in the North Atlantic, which makes remote sensing of aerosols difficult. Due to the long-range transport driven atmospheric dilution and relatively lower concentrations, Saharan mineral dust is not represented by particularly high AOD values, such as those associated with aerosols from biomass burning or local volcanic activity. However, several dust events, including the two unusual depositional episodes selected in this study, have been identified in Iceland based on the MODIS satellite products of the United States National Aeronautics and Space Administration (NASA), backward trajectories of the HYSPLIT model, and the NASA’s Modern-Era Retrospective analysis for Research and Applications, Version 2 (MERRA-2) dust column mass density data via NASA Goddard Earth Sciences Data Information Services Center (GES DISC) via Giovanni^93^ and BSC’s numerical model forecasts of dust products (AOD, dust load, and wet deposition) for the period 2008–2020^2^.

The systematic analysis of combined dark target and deep blue AODs at 0.55 µm for the land and ocean daily products of Terra MODIS (MOD08_D3 v6.1^94^), completed with backward trajectories of the HYSPLIT model, allowed the identification of the majority of events. Additionally, Area-Averaged of Dust Column Mass Density simulations of MERRA-2 (M2T1NXAER.5.12.4) were applied to verify the AOD-based observations and complete the dust episode database with cloud-obscured days. Outlying AOD values indicate an elevated dust concentration; therefore, backward trajectories at multiple altitudes were calculated for all AOD peaks to confirm the possible Saharan origin. The dust activity of each assumed source area was also confirmed using the visibility-reducing surface weather reports of the Naval Research Laboratory (https://www.nrlmry.navy.mil/aerosol/#aerosolobservations) at the end-points of the trajectories.

Aerosol v4.10 subtype vertical profiles obtained from CALIPSO (https://www-calipso.larc.nasa.gov/) were applied to provide another independent confirmation of the atmospheric presence of mineral dust over Iceland.

Dust load and wet deposition data of DREAM8 and NMMB/BSC dust numerical simulations were used to identify Saharan dust over Iceland and designate days of possible dust samplings^2,95^. The online multi-scale atmospheric dust model of the BSC provides daily dust load and deposition forecasts, which can also be used for the study area, even though the region was not part of the modelled domain before 2015. The movement of dust-loaded air masses towards Iceland could be clearly seen from the numerical simulations.

### Synoptic meteorology and HYSPLIT backward trajectories

Mean geopotential height and wind vector maps at 700 hPa were compiled to define the governing synoptic meteorological patterns by using the daily mean composite application of the Earth System Research Laboratory at the United States National Oceanic and Atmospheric Association (NOAA) (http://www.esrl.noaa.gov/psd/). To determine the dust transport pathways, 120–144 h backward trajectory analyses from the sample collection location were applied using the HYSPLIT model^96,97^. The meteorological input data for the synoptic calculations and trajectory model were gathered from the Reanalysis Project dataset^72^ of the NCEP/NCAR.

### Automated static image analysis

Direct optical image-based granulometric measurements of mineral particles provide a unique opportunity to determine the size and shape parameters of dusty material. The automated microscopic approach allows the characterisation of hundreds of individual mineral particles, thus providing the opportunity for a robust statistical analysis.

In this study, a Malvern Morphologi G3-ID advanced automated static image analyser was used. Approximately 7 mm^3^ of mineral material per sample was dispersed onto a flat glass slide with an instantaneous (10 ms) pulse of compressed air (4 bar) and a settling time of 60 s. Particles were scanned using a 20× magnification lens (0.025 µm^2^ per pixel resolution) attached to a Nikon eclipse microscope with z-stacking enabled (two focus layers were added above and below the initial focal plane, equivalent to a ± 13.5 μm z-height focus range).

Particle size and shape parameters of ~ 80,000 to 160,000 individual particles per sample were automatically recorded by the device software. Circle-equivalent (CE) diameter, aspect ratio, circularity, convexity, and solidity granulometric parameters were determined in this study. The CE diameter is calculated as the diameter of a circle with the same area as the projected two-dimensional particle image. The aspect ratio is the ratio of the particle width/particle length. Circularity is a proportional relationship between the circumference of a circle equal to the object’s area and perimeter. Convexity is the ratio of the convex hull to a particle’s perimeter, while solidity is the ratio of the area of the convex hull to the particle area. Aggregated and stacked particles were filtered by their certain parameters; particles with a circularity of < 0.6 were excluded from the granulometric characterisation. A cluster analysis was applied to determine the similarities of size and shape patterns in samples from different sources via a quantitative approach. Hierarchical cluster trees were created using the Euclidean distance pairs of the selected parameters.

A built-in Kaiser Rxn1 Raman spectroscope was used for the chemical analysis of particles. The spectra acquired using a 785 nm (< 500 mW) laser over 5 s were correlated to library spectra (BioRad-KnowItAll Informatics System 2017, Raman ID Expert) to identify quartz particles among the targeted sedimentary grains. The employed automated static image analysis with Raman spectroscopy has been previously successfully applied to identify Saharan quartz particles in the unique basaltic-calcarenite sequences of Fuerteventura in the Canary Islands^92,98^.

## Data Availability

All data needed to evaluate the conclusions in the paper are present in the paper.
