# Author Correction: Saharan dust and giant quartz particle transport towards Iceland

**DOI:** 10.1038/s41598-021-96399-0

**Published:** 2021-08-18

**Authors:** György Varga, Pavla Dagsson-Waldhauserová, Fruzsina Gresina, Agusta Helgadottir

**Affiliations:** 1 Geographical Institute, Research Centre for Astronomy and Earth Sciences, Budapest, Hungary; 2 Faculty of Environmental and Forest Sciences, Agricultural University of Iceland, Reykjavik, Iceland; 3 Faculty of Environmental Sciences, Department of Ecology, Czech University of Life Sciences, Prague, Czech Republic; 4 Institute of Geography and Earth Sciences, Faculty of Science, Budapest, Hungary; 5 Soil Conservation Service of Iceland, Hella, Iceland

Correction to: *Scientific Reports* 10.1038/s41598-021-91481-z, published online 04 June 2021

The original version of this Article contained an error in the spelling of the author Pavla Dagsson-Waldhauserová which was incorrectly given as Pavla Dagsson-Walhauserová.

The original Article has been corrected.

